# Incidence of Persistence and Recurrence of Differentiated Thyroid Cancer in Post-surgical Cases From a Tertiary Care Hospital in Dubai, United Arab Emirates

**DOI:** 10.7759/cureus.63555

**Published:** 2024-07-01

**Authors:** Mohammed H Al-Haideri, Malek Othman, Donia Ahmad, Maryam Alshamsi, Mazin Al Janabi

**Affiliations:** 1 Orthopaedics and Trauma, Rashid Hospital, Dubai, ARE; 2 Internal Medicine, Tawam Hospital, Abu Dhabi, ARE; 3 College of Medicine, Mohammed Bin Rashid University of Medicine and Health Sciences, Dubai, ARE; 4 Otolaryngology, Rashid Hospital, Dubai, ARE; 5 Nuclear Medicine, Mediclinic City Hospital, Dubai, ARE

**Keywords:** reoperation, anti-thyroglobulin antibodies, thyroglobulin, nuclear medicine, thyroid cancer, recurrent thyroid cancer, thyroidectomy, radioactive iodine therapy, papillary cancer of thyroid, differentiated thyroid cancer

## Abstract

Background

Despite the excellent prognosis of differentiated thyroid carcinoma, recurrence remains a major concern. However, the persistence of thyroid cancer post-thyroidectomy is not uncommon. We aimed to characterise patients who underwent re-operative surgery for differentiated thyroid carcinoma and analyse the percentage of re-operations that truly were for "recurrent" disease versus the management of persistent disease.

Methods

We conducted a retrospective review of the hospital database, analysing patients who visited the nuclear medicine department at Mediclinic City Hospital, a tertiary care hospital in Dubai, United Arab Emirates, between 2015 and 2022. The study included patients with differentiated thyroid carcinoma who underwent re-operations after total thyroidectomy. Recurrence was defined as the development of disease after a patient had undetectable thyroglobulin and negative radiological scans within one year of the first surgery. Cases were categorised as "recurrent", "persistent", or "unable to classify" in the event of missing data.

Results

Out of 836 patients diagnosed with differentiated thyroid carcinoma who visited the nuclear medicine department, 71 underwent re-operations. The mean age of these patients was 44.4 years (CI 41.7-47.0), of whom 78.9% were females. Almost half (46.5%) underwent re-operations within the first year, and 98.6% were diagnosed with papillary thyroid carcinoma. We were able to classify 63.4% of cases (n=45) as persistent disease, while 24 cases were categorised as "unable to classify". Only two cases met the criteria for recurrent disease.

Conclusion

The majority of cases previously classified as "recurrent" in differentiated thyroid carcinoma were found to be a persistent disease, possibly indicating inadequate therapy. Further research may be required to explore the reasons behind this eye-opening rate of disease persistence. This highlights an area for improvement in the management and future outcomes of differentiated thyroid carcinoma patients.

## Introduction

Despite the excellent prognosis of differentiated thyroid carcinoma (DTC), recurrence remains a major concern, with up to a 30% recurrence rate [1]. Sixty-six per cent of recurrence cases occur during the first 10 years after the initial management. Recurrence in the neck is serious and indicates a worse prognosis, including lethal outcomes [2]. Central neck recurrences are most commonly found in thyroid lymph nodes and thyroid remnant and, to a lesser extent, in the trachea and muscles [1]. Residual metastatic lymph nodes are by far the most common site for disease recurrence, highlighting the importance of surgical resection as a major determinant of the outcome [3]. This raises the question of whether recurrent DTCs are actual recurrence cases or simply persistent tumours due to insufficient management.

Lymph node metastasis, histologic variant, tumour size, extra-thyroidal extension, extra-nodal extension, male sex, and age above 55 years at the time of diagnosis are considered risk factors for recurrence [4,5]. Based on these factors, the American Thyroid Association (ATA) categorises DTC patients into low, intermediate, and high-risk groups, with higher recurrence rates in both intermediate and high-risk groups [6,7].

Several tools have been used to detect early DTC recurrence after therapy. Serum thyroglobulin (STg) measurement is the best tool to detect remnant thyroid tissue, including DTC [8]. Due to lower false-negative rates, current guidelines recommend measuring STg levels simultaneously with thyroid stimulating hormone (TSH) stimulation. Radiographic studies, including diagnostic radioiodine whole-body scintigraphy (DxWBS) and cervical ultrasonography (USG), are used for DTC recurrence detection. Cervical USG has a high sensitivity for detecting cervical metastases in DTC patients [9]. In a few DTC cases, even when STg levels were undetectable, USG detected neck metastases [10].

The ATA defines the absence of persistent DTC as follows: 1) absence of clinical evidence of the tumour, 2) no imaging evidence of tumour on DxWBS and cervical USG, and 3) undetectable STg levels with and without TSH stimulation (below 1 ng/mL and 0.2 ng/mL, respectively), in the absence of interfering antibodies [6]. Applying this definition in a clinical setting can help differentiate recurrent and persistent cases in patients undergoing a re-operation of what was previously considered "recurrent" DTC. Implementing this definition in clinical practice could identify areas for improving healthcare management, thereby enhancing therapy outcomes for patients with DTC.

## Materials and methods

**Figure 1:**
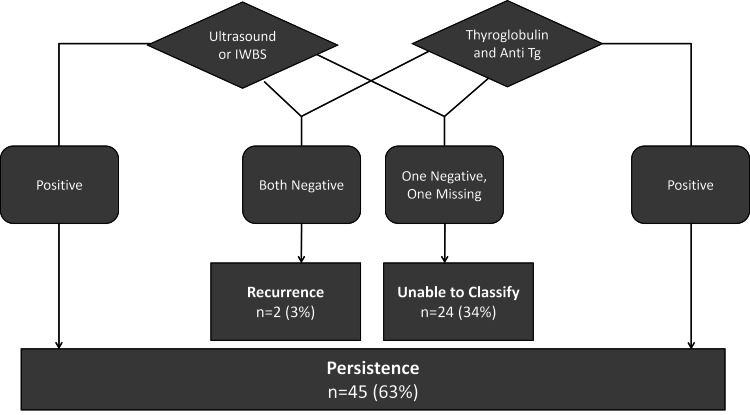
Flowchart illustrating the operation of the devised algorithm. IWBS: Iodine whole-body scintigraphy, Anti-Tg: Anti-thyroglobulin antibody.

Between 2015 and 2022, data were collected from patients presenting with thyroid disease. This study obtained approval from the ethics committee. The inclusion criteria comprised patients who underwent re-operative surgery for DTC during this period, specifically those who had previously undergone a complete thyroidectomy, whether through a one-stage or two-stage procedure involving a completion thyroidectomy, with or without cervical lymph node dissection. Exclusion criteria encompassed patients diagnosed with thyroid carcinoma types other than papillary thyroid carcinoma (PTC) and follicular thyroid carcinoma (FTC). Additionally, individuals with indications for neck surgeries unrelated to DTC were excluded. Following the ATA DTC guidelines, recurrence was defined as a disease developing after a patient had undetectable STg levels and negative radiological scans within one year of the first surgery [6]. An algorithm categorising patients into "persistence", "recurrence", and "unable to classify" was created based on ATA guidelines [6]. Figure 1 visually represents the algorithm's operation. Data from the year following the initial surgery were analysed to understand the reasons for undergoing a second surgery to manage DTC. Patients showing evidence of disease persistence through positive imaging or elevated STg levels within this period were classified as persistent cases. Recurrent cases were identified in patients lacking evidence of imaging or elevated STg levels within the first year post-surgery, while cases with insufficient data for classification were deemed unable to classify.

A total of 1,056 patients with thyroid-related diseases visited the nuclear medicine department at Mediclinic City Hospital, a tertiary care hospital in Dubai, United Arab Emirates, between 2015 and 2022, and among them, 836 were diagnosed with DTC. The inclusion criteria identified 71 patients (8.5% of total cases) who underwent re-operative surgery with some variation of cervical lymph node dissection following a complete thyroidectomy. Notably, after applying the algorithm (Figure 1), 45 patients (63% of these cases) met the criteria for disease persistence, while only two patients (3%) were classified as recurrent. The inability to classify 24 cases due to missing information, mostly from patients unable to provide proper documentation, who received treatment abroad, or who underwent their first surgery more than five years ago, highlights challenges in obtaining comprehensive patient data. Moreover, we believe the percentage of identified persistent cases would have been even higher with sufficient data to classify the remaining 24 patients.

## Results

The nuclear medicine department at Mediclinic City Hospital, Dubai, received 836 patients diagnosed with DTC over an eight-year period from January 2015 to December 2022. As one of the few well-established nuclear medicine facilities in the region, the majority of cases were referred from other hospitals, while some were diagnosed at our institute. Among the patients, 71 cases (0.08%) underwent two or more operations to remove tissues affected by DTC. In the initial surgery, only 23.9% of patients (n=17) had a specific neck lymph node dissection type. A total of 883 lymph nodes were removed during the initial surgery, with 376 (42.6%) found to be involved by DTC. Furthermore, 87.3% of patients (n=62) underwent Iodine ablation after the initial surgery. 

Sociodemographic and baseline variables

The mean age for patients was 44.4 years (CI 41.7-47.0), with the majority being females (78.9%, n=56). The most prevalent ethnicities were Emiratis (n=14) and non-Emirati Arabs (n=14), followed by Indians (n=13) and Filipinos (n=12). A total of 98.6% (n=70) were diagnosed with PTC, including two cases with concurrent FTC, as indicated in the initial surgery pathology report. Isolated FTC was observed in only one case. Among the 70 PTC patients, the classical variant was predominant (60%), while 13, five, and three patients had follicular, mixed, and tall cell variants, respectively. Hurthle cell PTC variant was associated with only one case, and the initial surgery reports were missing for six patients. Most initial surgeries (62.1%) were total thyroidectomy, while 17 cases had a specific node dissection type in addition to total thyroidectomy. One-side hemi-thyroidectomy followed by completion thyroidectomy accounted for 12.1% (n=8) of initial surgeries, and reports for the remaining five cases were missing. The location of initial surgery was unknown for the majority of cases (n=33) due to missing reports. However, 14 cases had bilateral thyroid lobe involvement, with 12 and 11 cases showing involvement of the left and right lobes, respectively. Only one case had isolated isthmus involvement in the initial surgery.

Time to re-operation

**Figure 2:**
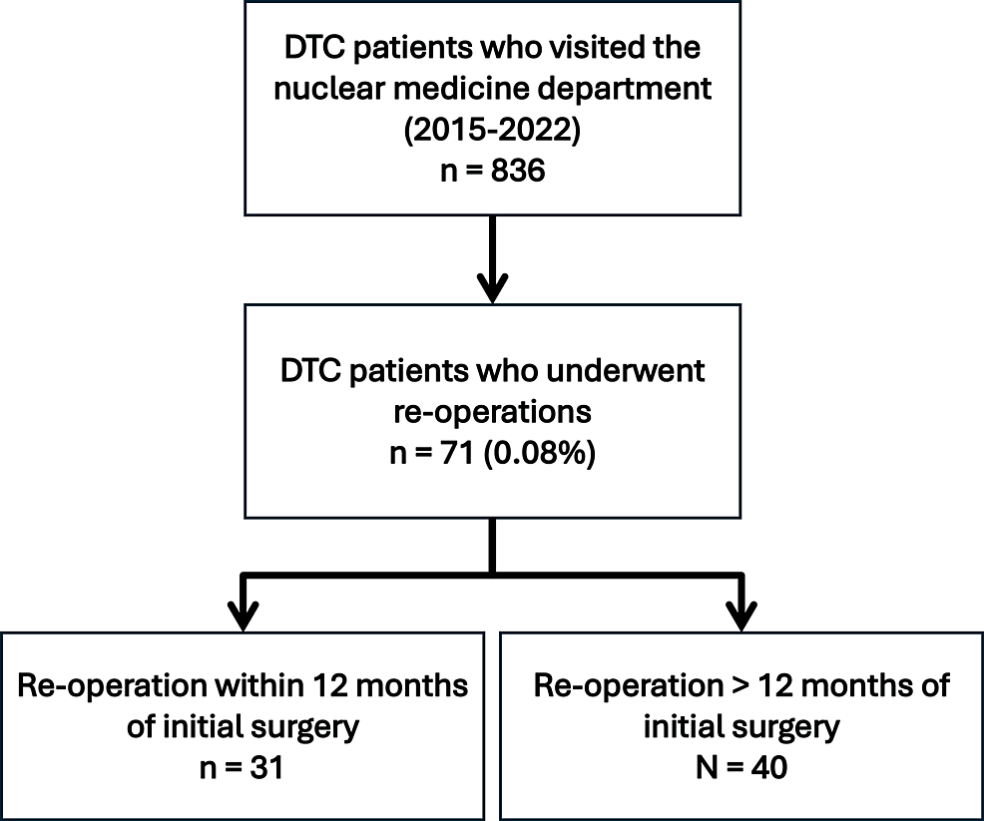
Study cohort. DTC: Differentiated thyroid cancer.

The median time between the initial surgery and re-operation was 13 months (CI 9.7-16.2). Approximately 43.7% of patients (n=31) underwent re-operation within one year of the first surgery (Figure 2).

**Figure 3:**
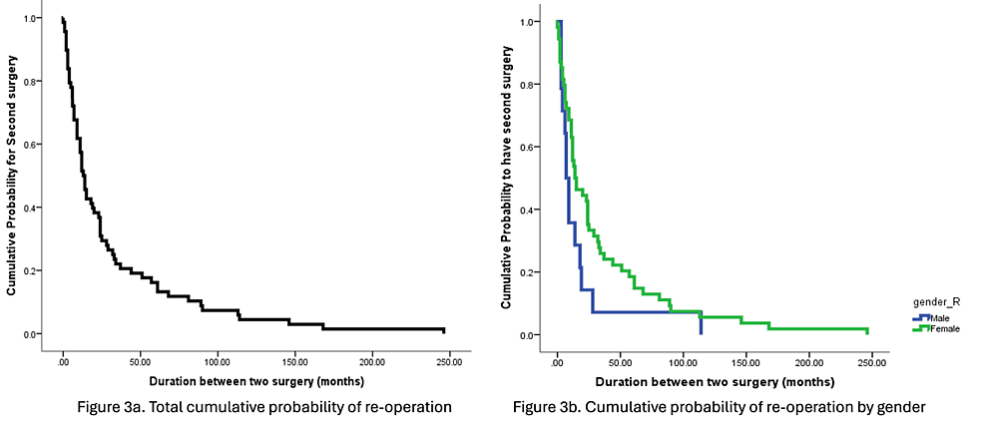
Kaplan-Meier estimator for time to re-operation.

The time intervals varied widely, with a range from 0.43 months (13 days) to 246 months (20.5 years) (Figure 3a). The probability of having a second surgery at six, 12, 18, and 24 months was 72%, 51.5%, 41.2%, and 30.9%, respectively. However, the probability of undergoing a second surgery within five years dropped to 0.118. Interestingly, the median duration to re-operation was seven months (CI 4.2-9.7) for males and 14 months (CI 4.3-23.6) for females (Figure 3b). Despite this difference, no significant association was found (p=0.084).

Persistence versus recurrence

Patients were classified into categories based on their levels of STg or anti-thyroglobulin antibodies (TgAb) and findings from USG or iodine whole-body scintigraphy (IWBS). The majority of patients (63.4%, n=45) were classified as having persistent disease. Among these 45 patients, 71.1% (n=32) had elevated STg levels without TSH stimulation, 86.7% (n=39) had an abnormal USG report, and 35.6% (n=16) had positive IWBS. Two cases had a negative USG finding with elevated STg levels, followed by abnormal IWBS findings. Only one case had a normal STg level (0.04 ng/mL) followed by an elevated STg level with TSH stimulation (2.67 ng/mL). All persistent DTC patients had abnormal radiologic findings, except one case with a high STg level (3.8 ng/mL) and a missing USG report. Only two patients (2.8%) met the criteria for recurrent DTC. These two patients were females, aged 62 and 44, and were Emirati and non-Emirati Arabs. The time to re-operation was 15 and 44 months, respectively. The STg level for both recurrent cases was 0.09 ng/mL, with undetectable TgAb and negative USG reports within one year of the initial surgery. Both cases were stage one PTC, one exhibiting the classic variant and the other the mixed variant. The initial surgery for both cases was total thyroidectomy without dissection, and both cases underwent Iodine ablation after the first surgery. The second surgery was a right selective neck dissection and bilateral neck dissection, respectively. However, 22 patients (33.8%) had missing follow-up reports and thus could not be labelled as persistent or recurrent DTC. These patients were put under the "unable to classify" category. 

Predictors of conclusion categories

In our study, we aimed to identify specific predictors of persistent DTC cases. However, no significant association was found for gender, ethnicity, PTC variant, tumour site, initial surgery, and re-operation types when comparing persistent cases to recurrent and unclassified cases (Table 1). Similar results were obtained when comparing persistent cases to recurrent cases.

**Table 1:** Distribution of sociodemographic and baseline variables by categories. UAE: United Arab Emirates, PTC: Papillary thyroid carcinoma, DTC: Differentiated thyroid carcinoma, TT: Total thyroidectomy, ND: Node dissection.

		Conclusion							P-value: persistence versus recurrence	P-value: persistence versus others
		Total number (%)	Persistence		Recurrence		Unable to classify			
			Count	Row (%)	Count	Row (%)	Count	Row (%)		
Gender	Female	56 (78.9%)	36	64.3%	2	3.6%	18	32.1%	0.65	0.76
	Male	15 (21.1%)	9	60.0%	0	0.0%	6	40.0%		
Ethnicity	UAE	14 (19.7%)	7	50.0%	1	7.1%	6	42.9%	0.759	0.388
	Non-Emirati Arab	14 (19.7%)	9	64.3%	1	7.1%	4	28.6%		
	India	13 (18.3%)	10	76.9%	0	0.0%	3	23.1%		
	Philippines	12 (16.9%)	9	75.0%	0	0.0%	3	25.0%		
	Asian countries	10 (14.1%)	4	40.0%	0	0.0%	6	60.0%		
	Western countries	8 (11.3%)	6	75.0%	0	0.0%	2	25.0%		
PTC variant (in PTC patients)	Classic	42 (59.2%)	28	66.7%	1	2.40%	13	31.00%	0.749	0.722
	Follicular	13 (18.3%)	8	61.5%	0	0.00%	5	38.50%		
	Mixed	5 (7.0%)	3	60.0%	1	20.0%	1	20.0%		
	Tall cell	3 (4.2%)	3	100.0%	0	0.0%	0	0.0%		
	Hurthle cell	1 (1.4%)	1	100.0%	0	0.0%	0	0.0%		
	Unknown	6 (8.5%)	1	16.7%	0	0.0%	5	83.3%		
DTC location on initial surgery	Bilateral	14 (19.7%)	14	100.0%	0	0.0%	0	0.0%	0.876	0.088
	Right	11 (15.5%)	9	81.8%	1	9.1%	1	9.1%		
	Left	12 (16.9%)	8	66.7%	0	0.0%	4	33.3%		
	Isthmus	1 (1.4%)	1	100.0%	0	0.0%	0	0.0%		
	Unknown	33 (46.5%)	13	39.4%	1	3.0%	19	57.6%		
First surgery type	Total thyroidectomy (TT)	41 (57.8%)	28	68.3%	2	4.9%	11	26.8%	0.736	0.804
	TT with selective node dissection (ND)	7 (9.9%	5	71.4%	0	0.0%	2	28.6%		
	TT with modified ND	10 (14.1%)	8	80.0%	0	0.0%	2	20.0%		
	Hemi-thyroidectomy followed by completion	8 (11.3%)	4	50.0%	0	0.0%	4	50.0%		
	Unknown	5 (7.0%)	0	0.0%	0	0.0%	5	100.00%		
Re-operation type	Selective ND	21 (29.6%)	13	61.9%	1	4.8%	7	33.3%	0.746	0.825
	Modified ND	30 (42.3%)	20	66.7%	1	3.3%	9	30.0%		
	Other	11 (15.5%)	8	72.7%	0	0.0%	3	27.3%		
	Unknown	9 (12.7%)	4	44.4%	0	0.0%	5	55.6%		

Table 2 displays TSH, TgAb, and STg levels after initial surgery with and without TSH stimulation. As in previous variants, no association was found between these levels and persistent disease. Although no significant association was found, post-surgical TgAb without TSH stimulation showed the lowest P-value (0.082). There was a noticeable difference in means between the persistence group and other categories.

**Table 2:** Post-surgical labs and conclusion categories. TSH: Thyroid stimulating hormone, TgAb: Anti-thyroglobulin antibodies, STg: Serum thyroglobulin, SD: Standard deviation.

		Conclusion						P-value
		Persistence			Other			
		Mean	SD	Valid N	Mean	SD	Valid N	
Without TSH	Post-surgical TSH	7.961	17.369	32	10.55	12.22	4	0.393
	Post-surgical TgAb	96.38	347.81	25	1.374	0.84	4	0.082
With TSH	Post-surgical TSH	91.37	34.89	17	100	0	3	0.765
	Post-surgical TgAb	93.97	277.7	11	18.2	29.8	3	0.456
	Post-surgical STg	106.3	131.35	14	80.03	53.7	2	0.7

## Discussion

Thyroid cancer is considered the most common endocrine disorder worldwide, and its incidence has been on the rise over the last decade, while mortality rates have remained steady or declined [11]. It is believed that the increase in incidence correlates with improved access to medical care and the identification of cases in earlier stages with improved management [11-13] DTC remains the primary and most diagnosed type of thyroid cancer [13]. It is essential to note that DTC carries an excellent five-year prognosis, with nearly 100% for localised disease, 98% for regional disease, and 53% for metastatic disease. However, a percentage of patients are found to have persistent/recurrent disease after initial treatment [14].

At present, most cases that require re-operations after an initial total thyroidectomy for thyroid cancer are considered recurrences. However, we questioned whether this is a true recurrence or rather a persistence of the initial disease. Following the ATA definition for the absence of persistent DTC, which includes the absence of clinical evidence of a tumour, no imaging evidence of a tumour on DxWBS and cervical USG, and undetectable STg levels with and without TSH stimulation (below 1 ng/mL and 0.2 ng/mL, respectively) in the absence of interfering antibodies within one year [6], we aim to identify whether a re-operation is indicated to achieve an optimal outcome for patients who initially presented post-operatively with negative cervical USG and undetectable STg levels or if these cases are presenting due to the persistence of the initial disease.

In a study conducted to identify the incidence of thyroid cancer in the United Arab Emirates (UAE) between 2011 and 2017, thyroid cancer emerged as the top endocrine malignancy and the second most common malignancy among females [15]. This pattern exhibited a notable female predominance, with Emirati patients showing a ratio of 4.86:1 and expat patients 2.47:1. The age of diagnosis typically occurred between the ages of 35 and 39 years [15]. The data revealed a 6.6% increase in thyroid cancer cases in the UAE during 2011-2017, equivalent to an annual rise of about 400 cases from 2011 to 2040. This information is crucial for identifying risk factors contributing to the rising incidence of thyroid cancer in the UAE, influenced by a combination of genetic, environmental, and demographic factors.

In the context of DTC patients undergoing re-operative surgery following initial treatment with total thyroidectomy, our study at a Dubai tertiary care hospital (Mediclinic City Hospital) revealed that 63.4% of cases were classified as persistent disease. Out of the total 71 re-operations, only two cases were considered true recurrences, characterised by undetectable STg levels and negative USG within one year. Among the remaining 69 cases, 46.5% underwent re-operation within the first year following initial surgery, emphasising the importance of adhering to ATA recommendations to minimise the risk of disease recurrence and metastatic spread. Surgery remains a crucial determinant of prognosis, with its adequacy significantly impacting outcomes. Complementary treatments like radioactive iodine (RAI) remnant ablation and TSH suppression play adjunctive roles. ATA guidelines advocate for total thyroidectomy as the first-line treatment for DTC, though recent guidelines allow for lobectomy or active surveillance in select low-risk patients. Post-operatively, RAI remnant ablation is considered for intermediate and high-risk patients [6].

A study analysing 4,292 patients with DTC who underwent total thyroidectomy with or without lymph node dissection between 1990 to 2016, excluding those with known distant metastases, conducted a multivariate analysis of risk factors predicting persistent or recurrent disease status. Independent predictors identified included male gender and advanced age, while tumour characteristics such as a follicular histotype and a higher tumour stage correlated with a stronger risk of persistent disease. Nodal metastases, especially at the regional lymph node (N1b level), were more frequent predictors of persistent disease compared to recurrent disease. This study highlighted that out of 498 cases of persistent DTC, 481 (97%) required post-surgical RAI therapy, contrasting with 35 out of 141 (24.8%) recurrent DTC cases. Persistent DTC followed a more aggressive pattern after diagnosis, leading to more patients requiring post-surgical RAI therapy [16]. These factors are crucial considerations in patient treatment, as they are associated with higher rates of unfavourable outcomes post-operatively regarding disease persistence. The study underscored the importance of laterocervical lymph node involvement (N1b) as an effective marker of negative prognosis and a predictor for DTC relapse [16]. Following initial treatment, long-term follow-up and monitoring are necessary to identify cancer recurrence, typically involving STg measurement, repeat neck USG and a radioactive scan. STg serves as an established biomarker for DTC recurrence/persistence, being secreted exclusively from thyroid tissue (normal or neoplastic). STg levels, particularly during the first year post-treatment, are sensitive and specific indicators of future recurrence [17]. If STg values exceed 2 ng/mL, ATA guidelines recommend DxWBSs to localise the source of STg and determine the need for RAI treatment [6]. Checking for TgAb in these patients is crucial, as higher values might mitigate disease persistence and can interfere with measured STg values [8,18].

STg levels play a crucial role in monitoring patients with thyroid cancer and can be assessed in two ways: through measurements taken when patients undergo stimulation of TSH and without any intentional stimulation. Unstimulated STg levels provide a baseline state, while stimulated STg levels encourage any remaining neoplastic tissue to produce and release STg into the bloodstream. These measurements are vital indicators in detecting the presence or absence of residual, persistent, or recurrent neoplastic tissue [19].

This crucial follow-up and investigation factor was lacking for a significant number of our "recurrent" cases. Due to the diversity of the patient’s healthcare backgrounds coming to our nuclear medicine department, ensuring quality and compliance with follow-up was a challenging variable to control.

Although STg serves as an effective surveillance biomarker against recurrence, it carries limitations, particularly in the presence of TgAb and in patients who have not undergone RAI ablation therapy. To address these challenges, neck USG emerges as a convenient tool for surveillance in such cases. It exhibits high sensitivity in detecting local and regional disease spread [6], aiding in the identification of features suggestive of recurrent disease, such as a hypoechoic mass in the post-operative thyroid bed, micro-calcifications, and marginal irregularity [20]. In a study that followed up low-risk patients with PTC to assess the role of neck USG in detecting lymph node metastasis during follow-up, it was observed that neck USG successfully detected nodal metastasis in 38 patients, seven of whom had a negative STg level. Notably, 50% of these patients had non-palpable and small lymph nodes measuring less than 1 cm. This emphasises the significance of neck USG in surveillance as per the ATA guidelines [6]. While STg levels remain a key marker for assessing the recurrence and persistence of DTC, an elevated value warrants additional investigation, including neck USG and/or DxWBS. In cases where imaging studies yield negative results despite elevated STg levels, MRI, or positron emission tomography (PET)/computed tomography (CT) can be performed to localise tumours and influence the management plan. MRI can readily identify nodal disease with high protein contents (colloid, STg, and blood products), aiding in the identification of extra-thyroidal invasion [21]. Current guidelines recommend MRI in high-risk DTC patients and for evaluating invasive recurrent disease when neck USG of nodal disease is inadequate [6]. Though the role of MRI as a first-line diagnostic tool remains controversial for post-operative follow-up, studies have highlighted its efficacy. A study conducted in 2010 compared the usefulness of MRI and DxWBS for detecting nodal metastases in patients with DTC and concluded that DxWBS was more specific [22]. However, MRI proved most effective in patients that had non-iodine-avid recurrences, exhibiting higher specificity and accuracy highlighting its utility in identifying and localising nodal metastasis. Another study conducted in 2009 evaluated MRI’s role in assessing patients with rising STg levels post-thyroidectomy and negative or indeterminate neck USG findings. The findings were focused on areas with restricted visibility on USG, such as retropharyngeal and parapharyngeal spaces. Nine patients meeting the criteria were identified. Among them, eight exhibited abnormal nodes in the retropharyngeal space, and one had involvement in the parapharyngeal space. The characteristics of these nodes varied, with six being solid, two showing complexity, and one being cystic. Notably, metastatic thyroid cancer was detected in five of these patients [23]. This emphasises the benefit of MRI in evaluating and conducting a comprehensive work-up for such patients.

Most importantly, we observed that the majority of re-operations in our cohort were conducted within one year of the initial surgery, prompting a re-classification of many patients from having a recurrent disease to rather a persistence of the initial disease. This sheds light on the challenge of achieving a disease-free status post-operatively, considering the impact of both unchangeable risk factors in DTC and the presence of modifiable factors. Addressing these changeable factors grants an opportunity to change our approach to the surgical aspect and shift our focus toward pre-operative preparations, including but not limited to lymph node mapping, adequate thyroidectomy, and emphasising the importance of a well-established follow-up treatment plan. However, our study carries certain limitations. Conducted retrospectively, it was limited by the availability of collected data in the system, making it challenging to classify patients accordingly, with respect to disease persistence or recurrence. This limitation hinders the identification of factors contributing to disease persistence and the pre-operative stage of the management plan. Consequently, distinguishing between recurrent and persistent disease becomes difficult. Another area of interest that is worth exploring would be intra-operative USG. This imaging modality is cost- and time-effective and can be utilised in the operation theatre with relative ease and minimises radiation patients are exposed to. Intra-operative USG is already routinely used in the surgical treatment of tumours in different centres worldwide. It has been showing promising results even when compared with preoperative CT scans. A recent study conducted in March of 2022 described the use of intra-operative USG during mediastinal lymph node dissections. It concluded that it helped increase the number of harvested lymph nodes and reduce the risk of intraoperative injury [24]. Similarly, another study conducted in 2006 on 512 patients with breast cancer revealed that intraoperative USG not only detected metastatic non-sentinel nodes in 95.7% of cases but also identified metastatic non-sentinel nodes in patients with false-negative sentinel node mapping, aiding surgeons in deciding whether proper axillary lymph node dissection was necessary [25]. Intraoperative USG was also utilised in the context of thoracic oesophageal cancer treatment. It concluded that intraoperative USG showed superior sensitivity compared with preoperative CT scans in detecting recurrent laryngeal nerve lymph node metastasis in patients with thoracic oesophageal cancer [26]. These findings in the literature hold promise regarding the use of intra-operative USG in the identification and treatment of DTC in the operation theatre, especially given the concern surrounding the effectiveness of current surgical practices in eradicating the disease and minimising persistence.

In summary, our observations reveal a significant number of patients undergoing re-operations for DTC, with a considerable proportion classified as having persistent disease due to positive post-operative findings of high STg values and/or abnormal neck USG. Several contributing factors, including but not limited to pre-operative, operative, and post-operative variables, lead to this conclusion. Working towards improving management at high-risk stages can enhance care for those with DTC, minimising the risk of recurrence and avoiding unnecessary interventions and reoperations.

## Conclusions

The majority of previously considered "recurrent" differentiated thyroid carcinoma (DTC) cases were found to be a persistent disease, possibly illustrating inadequate therapy. Further research may be required to explore the reasons behind this eye-opening rate of disease persistence. This highlights an area of improvement in management and future outcomes in DTC patients.

The persistence of DTC poses a significant threat to patient quality of life and disease prognosis. Extra measures must be taken to optimise the surgical aspect of treatment to improve outcomes and limit persistence. Possible improvements may include exclusively performing thyroid cancer operations at high volume centres specialised in thyroid surgery, the routine use of intraoperative USG imaging during thyroidectomies as well as the use of preoperative MRI imaging utilising its superior sensitivity in viewing soft tissue in head and neck when compared cervical USG imaging.
